# Dietary Fiber and Telomere Length in 5674 U.S. Adults: An NHANES Study of Biological Aging

**DOI:** 10.3390/nu10040400

**Published:** 2018-03-23

**Authors:** Larry A. Tucker

**Affiliations:** Department of Exercise Sciences, College of Life Sciences, Brigham Young University, Provo, UT 84602, USA; tucker@byu.edu; Tel.: +1-801-422-4927

**Keywords:** complex carbohydrate, ageing, lignin, whole grains, resistant starch, diet

## Abstract

The relationship between fiber intake and telomere length was evaluated using a cross-sectional design and an NHANES sample of 5674 U.S. adults. Another purpose was to test the impact of potential confounders on the association. Fiber consumption was measured using a 24 h recall and telomere length was indexed using the quantitative polymerase chain reaction method. Overall, the U.S. adults had low fiber intake (median: 6.6 g per 1000 kcal)—less than one-half the recommendation of the Dietary Guidelines for Americans. With age, gender, race, housing status, and misreported energy intake controlled, the relationship between fiber intake per 1000 kcal and telomere length was linear (*F* = 9.5, *p* = 0.0045). Specifically, for each 1 g increment in fiber intake per 1000 kcal, telomeres were 8.3 base pairs longer. Because each additional year of chronological age was associated with telomeres that were 15.5 base pairs shorter, results suggest that a 10 g increase in fiber intake per 1000 kcal would correspond with telomeres that are 83 base pairs longer. On average, this would equate to 5.4 fewer years of biologic aging (83 ÷ 15.5). With smoking, BMI, alcohol use, and physical activity controlled, as well as the other covariates, each 10 g increment in fiber accounted for telomeres that were 67 base pairs longer (*F* = 7.6, *p* = 0.0101), a biologic aging difference of about 4.3 years. In conclusion, significant fiber consumption accounts for longer telomeres and less biologic aging than lower levels of fiber intake.

## 1. Introduction

Dietary fiber has a significant impact on health and aging. Numerous studies show that individuals who consume high levels of fiber live longer and experience less disease than their counterparts [1,2]. The benefits are consistently inverse, dose–response, and apply to both men and women. As fiber intake increases, risk of disease and premature death decreases. In short, fiber consumption is an important part of healthy aging. 

Among the key recommendations of the recent Dietary Guidelines for Americans (2015–2020), individuals are encouraged to consume foods that are nutrient dense and “retain naturally occurring components, such as dietary fiber” [3]. The Guidelines define fiber as nondigestible carbohydrates and lignin that are intact in plants and present naturally in foods. According to the Guidelines, fiber can also be extracted from natural sources or synthetically manufactured and added to foods. The Guidelines recommend the intake of at least 14 g of fiber per 1000 kcal [3]. However, in the United States, intake is only about one-half that amount (see Table 1).

Fiber consumption is inversely related to a number of age-related killers. At least five prospective cohort studies have focused on fiber intake and total mortality, showing that as fiber consumption increases, mortality decreases [4,5,6,7,8]. Meta-analysis results indicate that for every 10 g increase in fiber consumption, risk of death decreases by 11% [2]. Moreover, in the same meta-analysis, when adults with high fiber intake were compared to those with low intake, mortality was 23% lower among those with high consumption [2].

Besides total mortality, diseases common to advancing age, such as heart disease, stroke, type 2 diabetes, breast cancer, and others are also less common among those with high fiber intake [9,10,11,12,13,14,15,16,17]. For example, in a 2015 meta-analysis by Wu et al. that analyzed 18 prospective cohort studies, coronary heart disease mortality was 17% lower in those with the highest fiber consumption compared to those with the lowest [15]. In a meta-analysis by Threapleton et al., risk of stroke was 7% lower for each 7 g of fiber eaten per day [14]. Moreover, Schulze et al. reported in a meta-analysis summarizing nine prospective cohort investigations that risk of developing type 2 diabetes was 33% lower in adults with high cereal fiber intake compared to those with low consumption [16]. A meta-analysis by Dong et al. that included 10 prospective cohort investigations indicated that breast cancer for the highest compared to the lowest fiber consumption was 11% lower [17]. Similarly, dose–response analysis showed that for every 10 g per day increment in dietary fiber intake there was a 7% reduction in breast cancer risk [17]. 

There are several pathways by which fiber consumption could decrease risk of disease and premature mortality. One is telomeres. Telomeres are nucleoprotein caps positioned at both ends of chromosomes, analogous to the caps that protect the ends of shoe laces. Each time mitotic cells divide, telomeres shorten and part of the telemetric DNA fails to replicate. Over time, cells undergo a limited number of divisions. Hence, the shortening of telomeres is a mechanism of biologic aging and a measure of the senescence of cells [18,19,20].

The length of telomeres is highly related to chronological age [20,21]. In fact, telomeres are sometimes called the molecular clock of cells [22,23]. As age increases, telomeres become shorter by about 15 base pairs per year [24,25]. Telomere length is also a key factor in the pathobiology of disease and premature mortality [18,19]. Individuals with shorter telomeres tend to have higher rates of cancer [26,27], heart disease [28,29,30], diabetes [31,32], osteoporosis [33], and other chronic conditions [18,19], including total mortality [34]. 

Factors other than chronological age also affect telomere length and, therefore, biologic aging. For example, adults who engage in high levels of physical activity have longer telomeres than their counterparts [21]. Obese individuals have shorter telomeres than normal weight persons [35], and smokers have shorter telomeres than nonsmokers [36]. Low sleep quality and quantity are also significant predictors of telomere length [37], as is low socioeconomic level [25].

Diet also seems to affect telomere length. Some foods and nutrients contribute to longer telomeres and therefore reduce biologic aging, whereas others account for shorter telomeres. For example, individuals who consume high levels of nuts and seeds tend to have longer telomeres than those with lower intakes [38], whereas in the Multi-Ethnic Study of Atherosclerosis (MESA), processed meat intake was inversely related to telomere length, signifying increased aging [39]. Using a case-control design, Hou et al. [40] found that fruit intake was positively related to telomere length in a study conducted in Poland, but in a large sample of U.S. adults, Tucker found that as caffeine consumption increased, telomeres decreased in length [24]. In multiple investigations, intake of fats and oils has also been associated with shorter telomeres [41,42]. Similarly, persons with higher intakes of gamma-tocopherol tend to have shorter telomeres than their counterparts [43]. Conversely, Marcon et al. showed that individuals with higher vegetable consumption have longer telomeres than others [44]. 

To date, few investigations have studied the effect of fiber consumption on biologic aging. Results from the Nurses’ Health Study, which investigated white women primarily and no men, indicated that among a variety of dietary factors, fiber intake was directly related to telomere length [45]. Furthermore, as shown in a 2017 review by Rafie et al., a number of other studies have examined the relationship between various foods, food groups, and eating patterns and telomere length [46]. Results have been mixed. Some of the foods and food groups have been good sources of fiber, such as whole grains, cereals, nuts, legumes, fruits, and vegetables. However, fiber intake specifically has rarely been associated with biologic aging [46]. 

Numerous investigations indicate that dietary fiber reduces risk of disease and premature death. Some of the health benefits associated with dietary fiber could be a result of the preservation of telomeres, or, in other words, reduced cell aging. To date, the relationship between fiber consumption and telomere length has received minimal attention, and the association has never been evaluated in a large sample representing men and women of the United States. Hence, the purpose of the present study was to determine the extent to which fiber intake accounts for differences in telomere length in 5674 randomly selected adults from the National Health and Nutrition Examination Study (NHANES). A secondary objective was to ascertain the effects of several potential confounding factors, including age, gender, race, housing status, misreported energy intake, smoking, physical activity, alcohol use, and body mass index, on the fiber and telomere relationship.

## 2. Materials and Methods

### 2.1. Sample

For a number of decades, the National Health and Nutrition Examination Survey (NHANES) has been conducted by the Centers for Disease Control and Prevention to estimate the nutritional status and health of noninstitutionalized civilians residing in the United States. To gather data, the survey utilizes a multistage probability sampling design [47]. During the first step of the sampling process, primary sampling units are randomly selected. These are mostly individual counties. During the second step, city blocks, or their equivalent, are sampled from the primary sampling units. For the third step, within each block, households are randomly selected. Finally, individuals residing in selected households are selected within age–sex–race sampling subunits [47].

National survey data are typically collected in two-year cycles. Measurement of leucocyte telomere length was performed only during two two-year cycles, the four-year period 1999–2002. The telomere data became available to the public in November 2014. During this four-year period, NHANES (1999–2002) oversampled a variety of subgroups to produce more precise estimates. The oversampled subgroups included individuals ages 60 and older, African Americans, Mexican Americans, persons 12–19 years old, and low-income individuals. All NHANES data are cross-sectional [47]. The NHANES (1999–2002) data sets that were examined in the present investigation, and others, can be accessed for free by the general public [48].

During the four years that telomere measurements were obtained, all participants 20 years of age and older were asked to give a DNA sample. A total of 7827 adults provided a useable DNA sample out of 10,291 eligible individuals (76%). To maximize confidentiality, individuals who were ≥85 years old were excluded from the sample because NHANES (1999–2002) recorded all of these individuals as 85 years old.

To be part of the present investigation, all participants were required to have complete data, including values for age, sex, race, housing status, misreported energy intake, body mass index, smoking pack years, physical activity, alcohol use, fiber intake, and telomere length. Included in the sample were 5674 adults residing in the United States—3005 women and 2669 men. Each NHANES (1999–2002) participant was required to provide written informed consent. The Ethics Review Board of the National Center for Health Statistics approved collection of the data and posting of the data files online for public use through the ethical approval code Protocol #98-12 [49]. 

### 2.2. Methods

For the present investigation, the exposure variable was total fiber consumption, expressed as grams per 1000 kcal consumed. The outcome variable was leukocyte telomere length. There were nine potential confounding variables: age, sex, race, housing status, misreported energy intake, smoking, physical activity, alcohol use, and body mass index.

#### 2.2.1. Telomere Length

According to NHANES [50], “the telomere length assay was performed in the laboratory of Dr. Elizabeth Blackburn at the University of California, San Francisco, using the quantitative polymerase chain reaction method to measure telomere length relative to standard reference DNA (T/S ratio), as described in detail elsewhere [25,51]. Each sample was assayed 3 times on 3 different days. The samples were assayed on duplicate wells, resulting in 6 data points. Sample plates were assayed in groups of 3 plates, and no 2 plates were grouped together more than once. Each assay plate contained 96 control wells with 8 control DNA samples. Assay runs with 8 or more invalid control wells were excluded from further analysis (<1% of runs). Control DNA values were used to normalize between-run variability. Runs with more than 4 control DNA values falling outside 2.5 standard deviations from the mean for all assay runs were excluded from further analysis (<6% of runs). For each sample, any potential outliers were identified and excluded from the calculations (<2% of samples). The mean and standard deviation of the T/S ratio were then calculated normally. The interassay coefficient of variation was 6.5%” [50]. The formula 3274 + 2413 × (T/S) was used to convert mean T/S ratios to base pairs.

#### 2.2.2. Fiber Intake

A 24 h dietary recall was administered by trained personnel using a computer-assisted interview [52,53]. Detailed information about all foods and beverages consumed during the 24 h period prior to the interview (midnight to midnight) was collected. Using the recall data, total fiber intake was calculated. Total energy intake was also measured, allowing fiber intake to be reported as grams per 1000 kcal consumed. Each diet interviewer was bilingual and a college graduate with a significant emphasis in the area of Food and Nutrition or Home Economics. The diet interviews were administered in a private setting in the mobile examination center (MEC). During the interview, a multipass format was used. Scripts were provided in the dietary recall system, which the Interviewers followed. The diet interviews were administered using a computer-assisted program that provided a standardized format. The diet recall included food probes that have been used successfully in previous United States Department of Agriculture (USDA) and NHANES surveys.

#### 2.2.3. Potential Confounding Variables

Statistical adjustments were made to control for differences in age, sex, race, housing status, misreported energy intake, smoking, body mass index (BMI), alcohol use, and physical activity. The following race/ethnicity categories were used in the investigation: Non-Hispanic White, Non-Hispanic Black, Mexican American, and Other Race, including Multi-Racial. Housing status (buying, renting, or other), another categorical variable, was evaluated to provide a general measure of social economic status. 

A measure of pack years was utilized to separate participants according to cigarette smoking. Pack years were calculated to represent participants’ use of cigarettes over time. The number of years the person was a smoker was multiplied by the number of cigarettes smoked per day and then divided by 20 [54]. Energy intake was calculated by using the 24 h recall findings. A fixed stadiometer with a moveable headboard was used to measure standing height [55]. A Toledo digital scale was used to measure weight while the subject was wearing only underwear, a disposable paper gown, and foam slippers [55]. BMI was calculated using the formula: weight (kg) divided by height (meters) squared [56].

Three groups were formed to define alcohol use: abstainers, moderate drinkers, and heavy drinkers. Adults who reported drinking no alcohol in the past year were defined as abstainers. Moderate drinkers were men who reported drinking more than 0 and less than 3 alcoholic beverages per day during the past year, or women who reported drinking more than 0 but less than 2 drinks per day over the past year. Heavy drinkers were men who reported drinking 3 or more alcoholic drinks per day over the past year, or women who reported drinking 2 or more alcoholic beverages per day over the past year.

Four statements were used to index physical activity, each referring to a different level of physical activity [57]. Subjects chose the phrase that best described their daily activities: “(1) You sit during the day and do not walk about very much; (2) You stand or walk about during the day, but do not carry or lift things very often; (3) You lift light loads or you have to climb stairs or hills often; (4) You do heavy work or carry heavy loads [57]”. 

When dietary assessments are self-reported, it is common for energy intake to be misreported [58]. The fiber consumption results of the present study would be affected if inaccurate reporting of energy intake occurred, because fiber consumption was expressed as grams of fiber consumed per 1000 kcal. Fiber consumption levels (per 1000 kcal) would tend to appear higher than they actually are if underreporting of energy intake happened. 

In this investigation, the 24 h recall strategy was used to assess diet. This strategy relies on self-reporting. Hence, some misreporting, particularly underreporting, likely happened. Several approaches to reduce the influence of misreporting are discussed by Mendez et al. [59]. In the present investigation, energy consumption was estimated and compared with self-reported values, and the calculated difference between the estimated and the self-reported values was utilized as a covariate to control for misreported energy intake in the regression models.

Specifically, using weight, height, gender, and age, resting metabolic rate (RMR) was estimated using the Mifflin RMR formula [60]. The physical activity (PA) variable discussed above was used to estimate physical activity level (PAL). The PA variable has four levels. It is an ordinal, categorical variable. Each level was assigned a PAL value: 1.45, 1.55, 1.65, and 1.75, respectively. RMR and PAL were multiplied together and the product was used to estimate total energy expenditure [58]. 

To assess validity, self-reported and estimated energy intake values were each correlated with BMI, after adjusting for differences in gender, race, and PA. There was not an association between self-reported energy intake and BMI (*F* = 0.6, *p* < 0.4634). However, estimated energy intake was strongly related to BMI (*F* = 1743.5, *p* < 0.0001), as it should be. 

### 2.3. Statistical Analysis

Each subject in NHANES (1999–2002) was assigned an individual sample weight [61]. The number of people in the United States represented by that sample person is reflected by each weight. The sample weights represent the unequal probability of selection, nonresponse adjustments, and modifications due to independent population controls [61]. Unbiased national estimates are produced when the sample weights are utilized as part of analyses. For the present investigation, the sample weights were based on diet records, which included the fiber intake data. 

Many scientists assume that the statistical power associated with NHANES investigations is high because of the large sample size. However, the multilevel, nested sampling approach of NHANES results in relatively few degrees of freedom (df). In the present study, analyses were conducted with 29 df in the denominator, calculated by subtracting the 28 strata from the 57 clusters.

In the Results section, frequencies are reported for categorical variables and means are given for continuous variables to help describe the sample. Standard errors are reported for the frequencies and the means. SurveyFreq was employed to calculate weighted frequencies, generalizable to the U.S. adult population, and SurveyMeans was used to develop weighted means that also represent outcomes for the U.S. population.

The main outcome variable for this cross-sectional investigation was leukocyte telomere length. Because the distribution deviated significantly from normal, the telomere values were log-transformed. Results were similar using the original telomere values and the transformed values. The exposure variable was fiber intake, expressed as total grams of fiber consumed per 1000 kcal. The SurveyReg procedure was used to conduct the analyses. With fiber intake and telomere length both treated as continuous variables, the magnitude of the linear relationship was evaluated. Additionally, the extent to which mean telomere lengths differed across categories of fiber intake was analyzed using linear regression. The fiber (per 1000 kcal) intake groups were based on sex-specific quartiles, with the middle quartiles combined: Low (25%), Moderate (50%), and High (25%). For women, the cut-points were Low, 0–4.9 g per 1000 kcal; Moderate, 5.0–9.9; and High, ≥9.9 g. For men, the cut-points were Low, 0–4.3 g per 1000 kcal; Moderate, 4.4–8.7; High, ≥8.7 g. To determine the extent to which the relationship between fiber consumption and telomere length was influenced by potential confounding factors, adjustments were made statistically using partial correlation. The least squares means procedure was employed to generate adjusted means. 

All *p*-values were two-sided and alpha was set at <0.05 for statistical significance. SAS version 9.4 (SAS Institute, Inc., Cary, NC, USA) was used to perform the statistical analyses.

## 3. Results

Individual sample weights were utilized so that the results are generalizable to the civilian adult population of the United States. Average (±SE) age of the sample was 46.5 ± 0.5 years, mean fiber consumption was 15.6 ± 0.3 g per day, and average fiber intake per 1000 kcal was 7.5 ± 0.1 g per day. Using estimated energy intake instead of self-reported, average fiber intake per 1000 kcal was 6.7 ± 0.1 g per day. Mean telomere length was 5826 ± 39 base pairs. Additional descriptive information for the sample is displayed in Table 1.

Linear regression showed that age was predictive of leukocyte telomere length (*F* = 598, *p* < 0.0001). The Pearson correlation was −0.44 (*p* < 0.0001). For each additional year of age, telomere length was 15.5 base pairs shorter, on average.

Descriptive information about each categorical variable is displayed in Table 2. For each subgroup, the number of subjects (*N*) is given in unweighted form, along with the percentage and the standard error of the percentage. The percentages displayed in Table 2 represent the weighted distribution of the sample. Attention should be focused more on the weighted percentages because they are generalizable to the civilian, noninstitutionalized adults in the United States.

With both fiber intake (grams per 1000 kcal) and telomere length treated as continuous variables, regression analysis showed that the relationship between the two variables was linear. After adjusting for differences in age, gender, race, housing status, and misreported energy intake, the association between fiber consumption (per 1000 kcal) and telomere length was significant (*F* = 9.5, *p* = 0.0045), with 29 degrees of freedom (df) in the denominator. Specifically, for each increase of 1 gram of fiber per 1000 kcal, telomeres were 8.3 base pairs longer, on average. After adjusting for all of the covariates simultaneously (age, gender, race, housing status, BMI, smoking pack years, physical activity, alcohol use, and misreported energy intake), the relationship remained linear and significant. For each increase of 1 g of fiber per 1000 kcal, telomeres were 6.7 base pairs longer, on average (*F* = 7.6, *p* = 0.0101).

Table 3 shows mean differences in telomere length after adjusting for age, gender, race, housing status, and misreported energy intake (Model 1). Model 2 displays mean differences in telomeres across fiber intake categories after adjusting for differences in all the potential confounding factors, including age, gender, race, housing status, misreported energy intake, BMI, smoking, physical activity, and alcohol use. The difference between those with Moderate fiber intake and those with Low intake was not significant. However, adults with High fiber consumption had significantly longer telomeres than those with Moderate or Low fiber levels (*F* = 3.8, *p* = 0.0357). On average, there were 75 base pairs separating those in the High compared with the Low fiber intake categories, with all of the potential confounders controlled.

Effect modification was tested with subjects stratified according to BMI levels. Focusing on individuals with BMI levels below 25 kg/m^2^, the relationship between fiber intake per 1000 kcal and telomere length was evaluated. After adjusting for differences in age, gender, race, housing status and misreported energy intake, for each 1 g increment in fiber consumption, telomere length showed a tendency to be longer by 10.0 base pairs (*F* = 3.95, *p* = 0.0563). With the sample delimited to overweight and obesity individuals, a BMI of 25 kg/m^2^ or higher, the fiber–telomere association was linear and significant (*F* = 4.89, *p* = 0.0350). Specifically, for each 1 g increase in fiber consumption per 1000 kcal, telomeres were 6.4 base pairs longer, on average, with age, gender, race, housing status, and misreported energy intake controlled. After adjusting for all the covariates, for each 1 g increase or decrease in fiber intake, telomeres remained 6.4 base pairs longer or shorter, respectively (*F* = 4.76, *p* = 0.0374). Similarly, with BMI delimited to obese adults—BMI > 30 kg/m^2^—the association between fiber intake and telomere length was linear and significant (*F* = 5.92, *p* = 0.0213), with age, gender, race, housing status, and misreported energy intake controlled. Specifically, among the obese, telomere length was 10.8 base pairs longer for each increment in fiber consumption per 1000 kcal. Likewise, after adjusting for all the covariates, the relationship remained linear and significant (*F* = 6.66, *p* = 0.0152), and telomeres were 11.9 base pairs longer for each 1 g increase in fiber consumption per 1000 kcal. 

## 4. Discussion

The main objective of the present investigation was to evaluate the association between dietary fiber intake (per 1000 kcal) and leukocyte telomere length in a randomly selected NHANES (1999–2002) sample of 5674 adults, representative of the U.S. population. A secondary purpose was to determine the extent to which a number of demographic and lifestyle factors influence the relationship between fiber consumption and telomere length. 

Results showed that total fiber intake was low, signifying that U.S. adults typically consume inadequate amounts of fiber. As shown in Table 1, median total fiber consumption was 13.6 g per day. The Dietary Guidelines for Americans (2015–2020) recommend that individuals consume at least 14 g per 1000 kcal [3]. When taking into account energy intake, the median intake in this U.S. sample was only 6.6 g per 1000 kcal—less than one-half the amount recommended by the Guidelines. Even adults reaching the 90th percentile in the present sample (12.8 g per 1000 kcal) fell short of the recommended standard. With self-reported energy intake replaced with estimated energy consumption, the median intake was even less at 5.7 g per 1000 kcal. 

In the present study, women had to consume about 10 g of dietary fiber per 1000 kcal or more and men had to eat at least 8.7 g per 1000 kcal to be assigned to the High fiber category—the upper sex-specific quartile. Given the significant advantage participants had when they achieved these levels of fiber consumption and attained the highest quartile, it appears that adults may not have to reach the Guideline recommendation of 14 g per 1000 kcal to receive some cellular aging benefits. 

Despite the overall low fiber intake, there was a significant linear relationship between fiber consumption and telomere length. The more fiber subjects consumed, the longer their telomeres tended to be. Specifically, for each 1 g increment of fiber intake per 1000 kcal, telomeres were 6.7 base pairs longer, on average, after adjusting for all the covariates. Given each additional year of chronological age was associated with telomeres that were 15.5 base pairs shorter, on average, it appears that a 10 g increase in fiber per 1000 kcal would be associated with telomeres that are 67 base pairs longer. On average, this would equate to 4.3 fewer years of biologic aging (67 base pairs ÷ 15.5).

Treating fiber intake as a categorical variable, quartiles were used to separate participants. Given there was a difference of 93 base pairs between the lowest and highest quartiles of fiber intake, with age, gender, race, housing status, and misreported energy intake controlled, the findings indicate that adults with High fiber consumption have a cell aging advantage of about 6.0 years over those with low intakes (93 ÷ 15.5). Adjusting for differences in all the potential confounders simultaneously (age, sex, race, housing status, misreported energy intake, smoking pack years, BMI, energy intake, alcohol use, and physical activity) attenuated the biologic aging benefit some. Specifically, after controlling for all the covariates, the telomere length difference was 75 base pairs. Hence, the cellular aging difference was approximately 4.8 years (75 base pairs ÷ 15.5)—a significant and meaningful spread. 

Calculating the biologic aging advantages or disadvantages of other lifestyle factors can add perspective to the fiber intake and telomere relationship. For example, research shows that for each 100 mg of caffeine consumed per day, adults have about 2.3 years of increased cell aging [24]. Conversely, consumption of nuts and seeds has been shown to predict less biologic aging. Specifically, for each 200 kcal of nuts and seeds consumed per day, adults have 1.7 years less biological aging [38]. Furthermore, consumption of sugar-sweetened soda appears to increase cell aging by 1.8 years for each 8 ounce serving per day [62]. Clearly, comparing fiber consumption to caffeine use, nut and seed consumption, and sugar-sweetened soda intake suggests that fiber intake plays a meaningful role in biologic aging differences.

Adjusting for differences in the lifestyle variables in addition to the demographic factors weakened the relationship between fiber intake and telomere length by about 15%. However, the association remained significant and meaningful. Apparently, only a small portion of the differences in telomere length across the fiber intake groups can be attributed to differences in smoking, BMI, alcohol use, and physical activity. 

In the present investigation, fiber intake was linearly related to telomere length. Higher levels of fiber consumption were correlated with longer telomeres, suggesting that a high-fiber diet may account for reduced biologic aging. Nevertheless, consumption of specific nutrients rarely occurs in isolation. In the present study, it is likely that individuals who consumed large amounts of fiber also ate significant quantities of fruits, vegetables, and whole grains [3]. Fiber intake is strongly linked to decreased risk of disease and premature death [2]. Likewise, consumption of fruits, vegetables, and whole grains is related to reduced disease and mortality [3,63,64]. Some of the health benefits associated with consumption of fruits, vegetables, and whole grains may be due to high levels of fiber intake. Similarly, some of the biologic aging advantages ascribed to high fiber intake may be a result of high intake of fruits, vegetables, and whole grains. Diets high in fiber and diets with significant amounts of fruits, vegetables, and whole grains go hand in hand. In the present study, part of the association between fiber intake and biologic aging could be a function of differences in fruit, vegetable, and whole grain consumption. 

To date, only one other large investigation has focused on the relationship between fiber intake and telomere length [45]. Telomere data were gathered via blood samples collected in 1989 and 1990. Using 2284 primarily white women and no men from the Nurses’ Health Study, Cassidy et al. determined that fiber consumption is directly related to telomere length. As expected, chronological age was inversely related to telomere length (*r* = −0.11, *p* < 0.0001), but the association was substantially weaker than the relationship between age and telomere length found in the present study (*r* = −0.44, *p* < 0.0001).

In an investigation that used rats as subjects, the effect of dietary fiber on telomere length was studied indirectly [65]. The rats were fed cooked meat at 15%, 25%, and 35% of their diets. Colonocyte telomere length was the outcome measure. After a month-long intervention, results showed that the telomere shortening effect of the high-meat diet was attenuated by resistant starch (insoluble fiber) intake. In short, dietary fiber reduced the unfavorable effects of the high-meat diet on the colon [65].

In 2017, Rafie et al. published a review of dietary variables as they relate to differences in telomere length [46]. A total of 17 studies were included in the review. Three of the studies indicated that the Mediterranean dietary pattern is associated with less cell aging. Similarly, five investigations showed that fruits and vegetables are favorably related to telomere length. Except for the Mediterranean diet pattern and fruit and vegetable consumption, findings were inconsistent. The majority of studies included in the review indicated no relationship between diet and telomere length. Only the Nurses’ Health Study by Cassidy focused on fiber intake specifically and telomere length [45].

A favorable relationship between telomere length and health outcomes is not automatic. Some investigations have revealed no relationship between markers of risk or disease and telomere length. For example, Njajou et al. studied 3075 men and women aged 70–79 years old and found no association between telomere length and overall mortality [66]. Additionally, mortality due to cancer and cardiovascular disease was not higher among those with shorter telomeres [66]. Similarly, Bischoff et al. uncovered no relationship between telomere length and survival in 812 adults, ages 73–101 [67]. Moreover, in a study conducted by the Telomeres Mendelian Randomization Collaboration et al., longer telomeres were actually related to increased risk of developing some cancers [68]. Consequently, the association between telomere length, disease, and mortality is still debated. 

In the present study, fiber intake accounted for significant and meaningful differences in telomere length. Why? Although the specific mechanism is not known, it is well established that telomere length and biological aging are strongly linked to inflammation and oxidative stress [69,70,71,72]. It is likely that the benefits of a high-fiber diet stem partly from this relationship. 

Diet can contribute positively or negatively to inflammation and oxidative stress, as shown by Zhou et al. [73]. Of the many dietary factors affecting inflammation and oxidative stress, fiber is significant. Numerous investigations show that as fiber intake increases, markers of inflammation and oxidative stress tend to decrease [10,74,75,76,77,78,79,80]. For example, in an investigation by Ma et al., which studied 524 individuals, both cross-sectional and prospective analyses indicated that fiber consumption protects against elevated C-reactive protein (CRP) levels [81]. Similarly, in a study of almost 5000 NHANES participants, King et al. showed that those with above-average levels of fiber consumption had significantly lower concentrations of CRP, even after adjustments for a host of demographic and lifestyle factors [82]. Additionally, in a sample of approximately 3500 older men, dietary fiber was inversely related to the inflammatory markers CRP and interleukin-6 [83]. It is likely that some, if not most, of the favorable association between fiber and biologic aging in the present study was a function of reduced inflammation and oxidative stress resulting from high levels of fiber consumption. 

An additional mechanism that could explain some of the shared variance between fiber intake and telomere length is blood glucose levels. As blood glucose concentrations increase, levels of inflammation and oxidative stress increase [84,85,86,87]. Fiber consumption slows the absorption of sugars, lowering blood glucose levels, insulin resistance, and risk of diabetes significantly [83,88,89,90,91,92,93]. Therefore, it is logical that high fiber intake could slow biologic aging and protect telomeres by reducing inflammation and oxidative stress caused by elevated blood sugar concentrations. 

The present investigation had multiple limitations. First, NHANES data are cross-sectional. Consequently, causal conclusions are unwarranted. Second, participants who reported high levels of fiber intake could represent unique individuals who have lifestyles that are healthier than others. Because of this threat, a number of potential confounding factors were controlled statistically, including age, gender, race, housing status, misreported energy intake, smoking pack years, BMI, alcohol use, and physical activity. These variables had little effect on the fiber–telomere association. However, other unmeasured factors could account for some of the association uncovered in this study. Third, the survey provided data about total fiber intake, but not insoluble or soluble fiber consumption. Information about fiber types would have strengthened the present study, but insoluble and soluble fiber data were not available and therefore could not be used in the present study. Finally, leukocyte telomere length can be altered by different diseases, particularly some cancers. Associations tend to be stronger for rarer cancers and at tissue locations with lower rates of stem cell division. The current study did not control for the presence of cancer or other disorders and, therefore, the results could be influenced by these diseases.

The present study also had several strengths. First, the sample was multiracial, large, and randomly selected using a multistage, probability sampling design. Hence, the findings are representative of the civilian, noninstitutionalized adult population of the United States. Second, a number of demographic and lifestyle variables were controlled statistically, minimizing their effect on the relationship. Results showed that the fiber–telomere relationship was independent of these factors. Third, a reputable laboratory was employed to measure leukocyte telomeres. Well-accepted methods were used to produce the telomere data, and chronological age was strongly related to telomere length, as it should be.

## 5. Conclusions

Total fiber intake (grams per 1000 kcal) was linearly related to leukocyte telomere length in a large sample of women and men representing U.S. adults. Additionally, with fiber intake divided into quartiles, adults with high fiber consumption had longer telomeres than their counterparts, suggesting significantly less biologic aging. A difference of 4.8 to 6.0 years in cell aging was found between those in the lowest compared with the highest quartiles of fiber intake. Overall, the present study highlights the risk of accelerated aging among U.S. women and men who do not consume adequate amounts of dietary fiber. The findings support the latest Dietary Guidelines for Americans (2015–2020) that recommend significant consumption of dietary fiber as part of a healthy diet.

## Figures and Tables

**Table 1:** Means, standard errors, and percentiles of the continuous variables (*n* = 5674).

			Percentile				
Variable	Mean	SE *	10th	25th	50th	75th	90th
Fiber (g)	15.6	0.26	5.6	8.9	13.6	20.0	27.7
Fiber (g per 1000 kcal)	7.5	0.11	3.2	4.6	6.6	9.4	12.8
Fiber (g per est. 1000 kcal)	6.7	0.11	2.4	3.8	5.7	8.5	11.8
Age	46.5	0.46	24.8	33.2	44.5	57.1	69.9
Energy intake (kcal)	2207	17	1102	1496	2042	2730	3513
Telomere base pairs	5826	39	5107	5383	5738	6172	6661

* SE is standard error of the mean. Fiber (g per est. 1000 kcal) is grams per estimated 1000 kcal, based on calculated resting metabolic rate (RMR) and physical activity level (PAL).

**Table 2:** Descriptive information for each categorical variable (*n* = 5674).

Variable	*N*	Percentage	SE
Sex			
Female	3005	52.8	0.6
Male	2669	47.2	0.6
Race			
Non-Hispanic White	2815	72.8	2.1
Non-Hispanic Black	1030	10.0	1.3
Mexican American	1400	7.4	0.8
Other race	429	9.8	1.8
Housing Status			
Buying	3727	69.5	1.7
Renting	1792	28.1	1.6
Other	155	2.4	0.4
Alcohol Use			
None	2209	34.4	2.9
Moderate	1745	32.4	1.9
Heavy	1720	33.2	1.4
Smoking Pack Years			
0	4594	78.1	1.1
1–10 years	649	12.5	0.7
>10 years	431	9.3	0.8
Physical Activity			
Sedentary	1397	24.5	0.8
Light	3052	51.2	0.8
Moderate	859	17.3	0.8
Intense	366	7.0	0.5
Fiber Intake per 1000 kcal			
Low	1285	25.0	1.0
Moderate	2715	50.0	1.0
High	1674	25.0	1.1
Body Mass Index			
Underweight	82	1.8	0.2
Normal	1609	31.1	0.7
Overweight	2064	34.8	1.2
Obese	1919	32.2	1.1

For each categorical variable, the “N” column represents the unweighted sample size (i.e., number of participants). The “Percentage” column represents the survey-weighted proportion of each subgroup within each categorical variable. Focus should be on the survey-weighted percentages because they represent the U.S. adult population. Some values may not sum to 100 because of rounding. The “SE” column represents the Standard Error of each percentage.

**Table 3:** Mean differences in telomere base pairs among Low, Moderate, and High levels of fiber intake in 5674 U.S. adults, after adjusting for potential confounding variables.

	Fiber Intake (Grams Per 1000 Kcal)				
Model	Low Mean ± SE	Moderate Mean ± SE	High Mean ± SE	*F*	*P*
Model 1	5800 ^a^ ± 51	5827 ^a^ ± 49	5893 ^b^ ± 46	4.5	0.0205
Model 2	5787 ^a^ ± 51	5804 ^a^ ± 51	5862 ^b^ ± 49	3.8	0.0357

Model 1: Tested the extent to which telomere means differed across Low, Moderate, and High fiber intake categories after adjusting for differences in age, sex, race, housing status, and misreported energy intake. Model 2: Tested telomere mean differences across Low, Moderate, and High fiber intake categories after adjusting for differences in all the covariates (age, sex, race, housing status, misreported energy intake, BMI, smoking pack years, physical activity, and alcohol use). ^a,b^ Means on the same row with the same superscript are not significantly different. Although there were 5674 participants, the *F*-tests were based on 29 degrees of freedom in the denominator because of nesting. The fiber (per 1000 kcal) intake groups were based on sex-specific quartiles, with the middle quartiles combined: Low (25%), Moderate (50%), and High (25%). For women, the cut-points were Low, 0–4.9 g per 1000 kcal; Moderate, 5.0–9.9; High, ≥9.9 g. For men, the cut-points were Low, 0–4.3 g per 1000 kcal; Moderate, 4.4–8.7; High, ≥8.7 g.
